# Supplementary material for: Yaws recurrence in children at continued risk of infection
Source: PLoS Negl Trop Dis. 2022 Mar 21;16(3):e0010197. doi: 10.1371/journal.pntd.0010197 (PMC8970528; doi:10.1371/journal.pntd.0010197)
Supplement: Attachment — Submitted filename: Yaws Paper Review .docx [file pntd.0010197.s002.docx]

**Review: Yaws recurrence in children at continued risk of infection**

**Berias et. al, 2021**

Overall, well written paper which adds significant evidence to the body of knowledge in the epidemiology of yaws and useful insights into recurrence of yaws.

**Abstract**

The abstract is well written and succinct. Captures all the essential results. Perhaps a brief sentence on the statistical methods employed in the abstract will be helpful

**Main Article**

**INTRODUCTION**

No major comments on introduction.

**METHODS**

a) Subjects
   a. There are variations in serological response to treatment, given that ulcer cure is not routinely confirmed RPR test, it is likely that a proportion of the patients classified as recurrent yaws may have had latent yaws.
   b. If there was clinical documentation of cure/remission (not serology) between episodes from the passive surveillance and whether this was considered in patient selection, the authors can provide that information as well.
b) Statistical analyses
   a. Generally, the use of the PWP (variance-corrected Cox model) for the regression analyses was appropriate including the clear assumptions and modifications the authors made. However, the authors may consider the use of a frailty/random effects model which accounts for the fact that some subjects are intrinsically more or less prone to recurrent events than others. This is particularly so with yaws because several factors (environmental, social, cultural, immunological etc) may affect the susceptibility to recurrent infections. If within-subject heterogeneity was accounted for in the PWP model, the authors can state that as well as that will address the issues that a frailty model will address.
c) Ethical consideration:
   a. No statement was made on ethical considerations for the study. The authors should provide some feedback on that.

**RESULTS**

a) Results are generally well captured with good flow.
b) A good addition to the results will be site of the recurrent ulcers (if data were collected). Did recurrent ulcers occur at different locations?
c) Page 3; Lines 103 -104 “***Treponema pallidum haemagglutination assay (TPHA) and RPR ≥ 1:8 are considered diagnostic of yaws”.***

However, a look at **Table 1** captures RPR titres of 1:08 – 1:16 in the classification of cases. Will be important to provide some explanation for this to readers and what considerations went into the categorization; 38% (Primary), 21% (First episode) of patients had RPR titres of **1:08 – 1:16** at primary episode.

d) Page 6. Figure 1.: It appears the annotation is wrong. “A” has be described as the flow diagram for the passive surveillance and “B” as active surveillance. Authors should re-look at this as it appears the annotations have been interchanged.
e) Brief mention of the other non-significant covariates from the regression analyses can be mentioned (RPR tire at primary episode, distance to hospital)

**DISCUSSION**

Page 11-*“Since all the cases received highly effective therapy under direct supervision, we assume that treatment failures were also very unlikely”.* – There various induvial level factors (host-immune response etc.)that can affect the serological response to treatment even in the context of highly effective treatment. Once ulcer cure was also not routinely confirmed in this study, this statement should be reconsidered.

*Reviewed by Rafiq Okine BSc MBChB MPH*
