# Supplementary material for: Yaws recurrence in children at continued risk of infection
Source: PLoS Negl Trop Dis. 2022 Mar 21;16(3):e0010197. doi: 10.1371/journal.pntd.0010197 (PMC8970528; doi:10.1371/journal.pntd.0010197)
Supplement: Attachment — Submitted filename: YawsRecurrence_re-submission letter and Responses.docx [file pntd.0010197.s003.docx]

**Camila González-Beiras**

Fight AIDS and Infectious Diseases Foundation

Badalona, Spain

cgonzalez@flsida.org

**Stuart Robert Ainsworth**

Associate Editor

PLOS Neglected Tropical Diseases

Dear Dr. Ainsworth,

On behalf of all co-authors, I would like to thank you for giving us the opportunity to submit a revised version of our manuscript **PNTD-D-21-01156**, entitled “Yaws recurrence in children at continued risk of infection” for publication consideration in PLOS Neglected Tropical Diseases.

As you will see in the point-by-point response to reviewers’ comments below this letter, we have addressed all comments and applied the suggested changes correspondingly. The comments were overall wise and we think they have improved the quality of our manuscript.

We hope the manuscript will now be suitable for publication in PLOS Neglected Tropical Diseases. We look forward to receiving your comments on our revised manuscript.

Sincerely,

C. González-Beiras

**Response to reviewers’ comments**

**Reviewer #1:** The objectives of the study were clearly tested. Hypothesis testing was not indicative for this study.

The study design was appropriate with good description of the study participants (some clarifications have been asked; included in the attachment)

Sample size was adequate. Few suggestions for statistical analyses have been included (see attachment)

No major ethical issues. The authors should however provide a statement on ethical considerations for the study

Minor Revisions required.

**Response:** Based on other comments, we have expanded the overview of the study design. This subsection is now entitled “Study setting and ethical conduct” and the information regarding informed consent has been moved to an independent paragraph and explained in more detail.

Regarding the PWP vs. frailty/random effects model approach to the statistical analysis, we thank the reviewer for his/her comment. We fully agree with your suggestion, however we estimated the models with sandwich variance estimators to account for the intra-subject correlation and stratified by the year of primary episode, allowing the baseline hazard function to differ throughout the entire follow-up period. The results of the crude and adjusted models were practically the same as those estimated by frailty-models (which we also estimated), but with a great reduction of the computational time. So, we chose not to use the frailty-models approach because the required computation time for the estimation of time-varying effects and interactions becomes unreasonable. In the revised version of the manuscript, we have added that the models were built using a robust variance estimate to account for the first year of the index episode.

**Reviewer #2:** (No Response)

**Reviewer #3:** - The hypothesis is not clear stated in the background. The background misses stating the link about the knowledge gap on immunity to re-infection and how this study (estimating recurrence with these particularly methods) can contribute to it.

Explaining in the background, that these first estimations of recurrence (as a first step) can start pointing towards the type of immunity (saying that you will use of molecular methods), while giving clear recommendations on the type of surveillance and follow up needed (in the discussion-recommendations), may clarify the justification of conducting this study and its added value.

**Response:** We thank the reviewer for this comment. In paragraph 2 of the background, we have extended the points made about what is known about acquired immunity to T.pallidum in humans and adults and also how better understanding recurrence is a first step to exploring acquired immunity to infection.

- The appropriateness of the study design could be better clarified, by explaining why these two methods are chosen (i.e. in the absence of a formal surveillance system), and how they correlate to each other (in time, in target study population), and the added value of putting both approaches together in a study.

**Response:** We agree that the double approach used in our study may hamper understanding if it is not well introduced. In the revised version of the manuscript, we have rephrased the first subsection of Methods and expanded the information provided by explaining the rationale of using two approaches.

- The study population is not clearly mentioned (and should be specified for the two surveillance approaches included). The study setting mentions Lihir island, but in the Passive surveillance description, it is only mentioned the Lihir Medical Centre (LMC) consulting patients, so it should be described which is the target population of LMC in comparison with Lihir population (i.e. also if there are any other health posts-centers in the island which were treating ulcers during the study period? and if different characteristics in access), if the treatment was for free for everybody (or certain age groups), etc. All this orients towards accessibility, and potentially completeness-representativeness of the passive surveillance data and its interpretations.

**Response:** The LMC is the only hospital in Lihir island. Aside from this center, the island has other aid posts that can treat yaws clinically but cannot perform serological tests. As described in the methods section (Passive surveillance), a positive serological test was required for defining the case as recurrent. Therefore, all recurrent yaws identified in the island and meeting the serological definition for recurrence were captured by the electronic health records of the LMC. In the original version of the manuscript, we acknowledged (limitations paragraph) the possibility that recurrences treated in peripheral aid posts escaped from our analysis, thus underestimating the actual burden of yaws recurrence. In the revised version of the manuscript, we have provided more information regarding the catchment population covered by the LMC and the presence of aid posts without capacity for serological testing of yaws (Methods, Overview of Study setting and ethical conduct. Methods, Passive surveillance).

--------------------

**Reviewer #1:** Analysis plan and results clearly presented.

Quality of the figure (flow diagram) provided should be improved

Minor revisions requested (See detailed comments in attachment)

**Response:** We thank the reviewer for noticing the wrong assignment of A and B panels in figure 1 legend. We have amended it, increased the font size for more clarity, and corrected a mistake noted by another reviewer regarding the box contents.

Regarding the minor revisions requested in the attachment:

We have included statistical information in the abstract.

We have revised the text regarding the definition of recurrent yaws, making more explicit the condition of “cutaneous ulcer” for being considered a recurrent episode (i.e., thus excluding latent yaws), and how an ulcer was documented.

Regarding the ulcer location, we only have this information for the active surveillance, which corresponds to a small sample and does not provide a remarkable insight.

In Table 1, we have changed 1:08 for 1:8 to prevent confusion and added a footnote indicating that the row is intended to show the sample distribution across categories.

The variables without contribution to the risk of recurrence have been highlighted in-text.

We have explained our hypothesis of low likelihood of treatment failures better and included a reference supporting the effectiveness of azithromycin.

**Reviewer #2:** (No Response)

**Reviewer #3:** - Clarity of the results:

In the passive surveillance, are there any differences among patients with positive and negative serology (in terms of demography, number of ulcers, distance to centre)?

**Response:** We agree that these data would provide interesting insights to interpret our results beyond the main objectives of our work. Unfortunately, the information collected during the passive surveillance was limited, and we cannot perform adequate analyses in this regard.

- Clarity of figures and tables:

Title of figures and table should be self-explanatory (at this point they miss to inform about which how data was collected (if from passive-active or both), from where, when, etc)

To specify in the title of Figure 1 that relates to both active and passive surveillance (to clarify since it is inserted in the Passive surveillance section)

In Figure 1, the A and B in the graph (A is active surveillance) does not correspond with the legend (A is passive)

In Figure 1, in the Diagnoses of cutaneous ulcers from medical records, there is a group of patients who have PCR (not clear why), since in the section of the Methods only serological methods are defined in the Passive surveillance.

**Response:** We agree that table titles were not self-explanatory. We have rephrased all titles and increased their accuracy.

We also thank the reviewer for noticing the mistake in Figure legend 1, which has been amended. We have also fixed an inconsistency with figure boxes.

Regarding PCR availability as selection criteria, it was required for defining molecular recurrence in individuals included in the active surveillance analysis, as it was described in the methods section (active surveillance subheading). Now that the assignment of panels A and B (as well as text in boxes) in figure 1 have been corrected and boxes, the corresponding methods are more clear.

--------------------

**Reviewer #1:** The study has significant public health indications and offers good evidence on the need for follow-up of yaws cases. The conclusions are valid.

Some suggestions have been made with respect to study limitations (see attachment)

Minor revisions required

**Response:** as suggested, we have rephrased the statement regarding the likelihood of treatment failure and provided a reference to support it.

**Reviewer #2:** (No Response)

**Reviewer #3:** - The main conclusion stated is supported by the findings from both surveillance systems.

- Further details on the limitations of using such medical records as surveillance system should be mentioned (i.e. only completeness already mentioned, not any other of the surveillance attributes which may affect the number of recurrent consultations).

**Response:** In the limitation sections, we have expanded the scenarios in which cases may remain unnoticed.

- The authors suggest absence of full protective immunity, but they could explain which methodologies are recommended to properly confirm this argument.

**Response:** We have added a section within the discussion highlighting that looking for evidence of re-infection with strains which are identical by whole genome sequencing (not just MLST) would add further weight against the development of full protective immunity.

- The public health relevance of the recommendation (conducting active follow up) is there, but it could be better integrated in the public health context (not as an isolated recommendation). For instance, it could be mentioned under which strategy (in the context of eradication plan) is recommended the active follow up for this/similar settings (i.e. active follow up embedded on a Lihi passive surveillance with ulcers as weekly notifiable disease? Active follow up under weekly or monthly active surveillance? Active follow up after regular MDAs?).

**Response:** In the revised version, we have expanded the benefits and suggested strategy for including this assessment in routine practice (Discussion, Conclusions paragraph). Currently, yaws surveillance focuses on the identification of azithromycin resistances. However, the lack of specific assessment of recurrences leave many of them unnoticed. The specific assessment of recurrences would allow identifying contacts and plan interventions beyond the case assessed (particularly in individuals returning from other environments, in which a recurrence may indicate incidence rebound in the area).

--------------------

**Reviewer #1:** Minor revision required (see reviewer's attachment)

**Response:** responses to minor comments in the attachment have been provided right after the first comment of the reviewer.

**Reviewer #2:** (No Response)

**Reviewer #3:** Minor modifications:

- To clarify which diagnostic codes were included under the passive surveillance – so it is possible to ensure the quality of the case definition used for the objective of the surveillance

**Response:** ICD-9 codes screened in the medical records have been included in the methods section (Passive surveillance)

- Accessibility is reflected in the analysis as Km to the health center in the passive surveillance analysis. But there are other aspects related to accessibility which are not considered. For instance, it would be useful to know whether treatment was offered for free, and any information on the type of care provided (24 hours service, etc), acceptability by population of the healthcare provided.

**Response:** We agree that further details regarding ease of access to healthcare resources in general would provide the reader with a more accurate picture of healthcare delivery and scope in Lihir Island. Unfortunately, the lack of detailed information at individual level prevented adjusting our analysis for differences between villages. We have included this information in the first paragraph of the methods section (Overview of Study setting and ethical conduct).

- For the active surveillance data– It would be useful to describe (or refer to in the text – if published elsewhere) how the active finding was done (so reader can understand how systematic was the population targeting and the coverage).

**Response:** The active surveillance of yaws recurrences was encompassed in longitudinal study described in detail in reference 1. We have included a brief summary of the methods after citing it.

- It is not clear how a new episode of PCR-confirmed was defined. Was clinical remission after treatment followed, or any other time variables?

**Response:** We agree that the previous text was unclear in this regard. We have explicitly mentioned the clinical remission before a PCR-confirmed new case for confirming recurrence. Within the active surveillance, all patients were followed up periodically; therefore, we could confirm the previous clinical remission.

- The use of same recurrence criteria for the case definition in the active and passive surveillances should be highlighted in the methods (and not only at the results section, at the end of the active surveillance system). For clarity, it could be considered to have a definitions sub-section in the methods section.

**Response:** The criteria used for recurrent disease are not identical in the Passive and Active Surveillance components. This is because molecular (i.e., PCR) testing was only available in the active surveillance component. As outlined in the methods for Passive Surveillance, we defined recurrence as “experienced a recurrent episode of cutaneous ulcer that met one of the following criteria of RPR titre change compared with the index episode: (1) 4-fold or greater titre increase within the 12 months following treatment or (2) absence of 4-fold or greater decline between 12 and 96 months after treatment. “ We have added a new figure to help illustrate this.

In active surveillance we defined a recurrence as:

“We defined molecularly confirmed recurrence when an individual had a new episode of PCR-confirmed yaws after clinical remission in the previous survey”

We discuss this difference already in the limitations where we state

“Our passive case detection analysis is also based only on serologically confirmed yaws which is not completely reliable. However, our findings are supported by our active case finding exercise where molecular confirmation was used.”

Notice that each analytical approach was used to address different objectives (as now explained in the first subsection of methods). Therefore, we do not mix definitions or recurrence criteria for a given objective.

--------------------

**Reviewer #1:** Overall, well written paper which adds significant evidence to the body of knowledge in the epidemiology of yaws and useful insights into recurrence of yaws.

**Response:** The thank the reviewer for his/her appraisal of our work.

**Reviewer #2:** Comments:

1. On the end of page 1/beginning of page w, the sentence beginning “studies in experimental models” is a bit confusing; can you clarify what “acquired protection to T. pallidum can develop in untreated animals and persist after treatment” means? What is meant by “acquired protection” – were they protected after a challenge? I think the next sentence explains it but it may be more clear just to drop that first sentence and expand on the theme of the paragraph – is it that there is a conflict between experimental models and humans in the generation of protective immunity?

**Response:** As the reviewer notices, the pre-clinical evidence cited in the introduction was gathered in challenge experiments. We have modified the sentence to make this clearer. As neither animals nor humans appear to be completely protected on re-challenge we believe the animal and human data are broadly concordant.

2. At the end of that same paragraph the sentence “humans may experience multiple episodes of syphilis and yaws, suggesting that immunity is either transient or potentially strain-specific” seems fairly limited in the interpretation of multiple infections. Even long-lived immune responses many not offer sterilizing immunity (see recent serological data on trachoma where kids get multiply infected yet have good antibody responses).

**Response:** We have reworded this sentence to make clearer the possibility that immunity may be non-sterlisizing.

3. Methods: Is it standard to have a definition of RPR titer >=1:8 as serological confirmation of yaws? Marks et al Lancet Global Health PMCID: PMC7116878 used an RPR titer of 1:2 as evidence of seroconversion, so it would be useful to give some justification for this decision. The discussion briefly calls this a high-titer cutoff (although the Marks et al paper seems to use 1:16 as a high titer cutoff – it would be helpful to have consensus on what RPR titer constitutes high titer).

**Response:** In the work by Marks et al., an RPR titer cutoff of 1:2 was used to define seroconversion. Titers ranging from 1:2 to 1:4 can indicate seroconversion caused by a previous exposure or very incipient and mild infection and, in some cases, these titers may remain for life (serofast). However, an active infection with clinical manifestations is typically associated with higher titers. Therefore, we established the 1:8 cutoff to prevent incorrect assignations of etiology of active ulcers. In the methods section, we added the term “active” to address this nuance.

4. Results, paragraph 1: 103/1486 does not = 60.7%. Assuming there is a typo somewhere here?

**Response:** We thank the reviewer for noticing this error, which has been corrected. Indeed, this was a typo. The correct percentage is 6.9%, rounded to 7 in the discussion section.

5. I am confused by the flow of Figure 1 – there seems to be an increasing number of people in the flow. IFor example, why is the flow going from a smaller number of patient with PCR available (50) to a larger number of patients with recurrent episodes (1246) with >4000 people excluded?

**Response:** The assignment of active and passive surveillance panels in Figure 1 legend were reversed. The reviewer is also right regarding the mistaken composition of boxes in the two figures (i.e., the passive surveillance panel mistakenly combined boxes from the two types of surveillance). We thank the reviewer for noticing these errors, which have been amended.

6. Discussion. I am a little confused by the actual conclusions about the recurrence of yaws. Due to the error I noted above it is not clear what % of cases seen were recurrent yaws in the passive case detection section. In the active case finding section, 8/12 PCR-confirmed in the index ulcer were PCR_confirmed as having recurrent ulcers. But the discussion says “A low ― albeit substantial (7%) ― proportion of patients with yaws had serologically confirmed recurrent yaws.” Does that mean the original numbers – 103/1486 – were correct and the 8/12 in active case finding was an anomaly? I am not seeing that you commented on this in the discussion. Is there an advantage to either approach?

**Response:** As explained before, the composition of the former figure 1 was wrong and mixed up boxes of the two panels. We have revised the figure, which now shows clearly that we identified 1486 yaws patients in our retrospective screening of medical records, ow which 103 (7%) had experienced recurrent yaws episodes. Regarding the double approach, we have included a rationale (and aims) for the two approaches in the methods section (overview of study setting and ethical conduct.

7. Discussion: you say that treatment failure is highly unlikely, but could you support that with a reference or data from one of the studies that looked at that? I know that I could look it up but your argument will be stronger with just a sentence with data to support it.

**Response:** We have added the reference of a RCT showing that single-dose of oral azithromycin was highly effective, and non-inferior to benzathine benzylpenicillin for the treatment of yaws.

8. Are you surprised at the finding: ‘Nearly half of individuals with recurrent yaws (43%) were children aged 15 years or younger.”? I admit that I am having trouble deriving this based on the Tables, which suggest the majoring of recurrent infections were in <15 year olds.

**Response:** This was a mistake; thank you for noticing it. We have re-written this to align with the table

**Reviewer #3:** The study presents data on recurrence yaws in a well-followed population, through the use of two distintictive data collection approaches. It is useful data to share with public health institutions working with yaws in PNG and other setttings.

The study is well written and the efforts to bring further understanding on the epidemiology of yaws in Lihir island with existing data are meritable. However, it is no very clear whether looking at retrospective medical registries is the most appropriate method to properly assess recurrence. It is my understanding that the registry was not set up with the objective to monitor cases or recurrence, so the registry should be further explained (i.e. for example in terms of attributes of the surveillance system - see CDC, ECDC surveillance system evaluations). The choice of these two methods, how they complement each other, and their weaknesses for this objective (and the point discussed on fully protective immunity) can be further explained. Thus, it can be better assessed about the methodological implications of measuring recurrence through it.

**Response:** As suggested (also in response to previous comments), we have included a brief rationale for the choice of this double approach of passive and active surveillance (methods > Study setting and ethical conduct) and a more detailed explanation of the hospital registry, including how likely was a yaws case to be diagnosed out the Lihir Medical Center. In our discussion section, we address the limitations of this methodology.

In terms of recommendations, further reflection on which kind of routine surveillance system would be needed for yaws eradication in the country and in the island would be welcome. Considering the previous work on yaws in the island, authors could propose and advocate on how active follow up could be integrated in this setting.

**Response:** The need for a more specific explanation about the suggested changes to current surveillance practices in the area was also highlighted in previous comments. In the revised version of the manuscript, we have expanded the conclusions paragraph in this regard.

**Reviewer #3:**

Details on ethical procedures are missing, mainly related to the consent process. The information provided in the study says that “all participants provided informed consent” but it should specify :

- The different consent process for the outpatient clinic participants and for the active surveillance participants. Was informed consent avaliable for conducting a research from the patients included in the passive surveillance data? In case not, it should be specified and whether this has been specifically approved by the local ethical review or exempted from full review.

- To specify how the consent to conduct research was processed for participants below 18 (in both surveillances) – was the caregiver providing with such a consent? Or was the legal responsible of the children. All this should be justified according to PNG law

- Whether consent was oral or written, and in which language was provided.

- Was assent obtained from children to participate (if so, from each age - according to PNG law)?

If those details are included in other publications from this data, please refer to them

**Response:** in the revised version, we have expanded the first subheading of the methods section and included a paragraph with further details on the ethical conduct of the study.
